# Ultrasound–Vortex-Assisted Dispersive Liquid–Liquid Microextraction Combined with High Performance Liquid Chromatography–Diode Array Detection for Determining UV Filters in Cosmetics and the Human Stratum Corneum

**DOI:** 10.3390/molecules25204642

**Published:** 2020-10-12

**Authors:** Fang-Yi Liao, Yu-Lin Su, Jing-Ru Weng, Ying-Chi Lin, Chia-Hsien Feng

**Affiliations:** 1 School of Pharmacy, College of Pharmacy, Kaohsiung Medical University, Kaohsiung 80708, Taiwan; pulu9@hotmail.com (F.-Y.L.); yclin@kmu.edu.tw (Y.-C.L.); 2 Department of Fragrance and Cosmetic Science, College of Pharmacy, Kaohsiung Medical University, Kaohsiung 80708, Taiwan; aitian897@gmail.com; 3 Department of Marine Biotechnology and Resources, National Sun Yat-sen University, Kaohsiung 80424, Taiwan; jrweng@mail.nsysu.edu.tw; 4 The Ph.D. Program in Toxicology, College of Pharmacy, Kaohsiung Medical University, Kaohsiung 80708, Taiwan; 5 Institute of Medical Science and Technology, National Sun Yat-sen University, Kaohsiung 80424, Taiwan; 6 Department of Medical Research, Kaohsiung Medical University Hospital, Kaohsiung 80708, Taiwan

**Keywords:** UV filters, ultrasound–vortex-assisted dispersive liquid–liquid microextraction, bio-derived solvent, human stratum corneum, cup method

## Abstract

This study explores the amounts of common chemical ultraviolet (UV) filters (i.e., avobenzone, bemotrizinol, ethylhexyl triazone, octocrylene, and octyl methoxycinnamate) in cosmetics and the human stratum corneum. An ultrasound–vortex-assisted dispersive liquid–liquid microextraction (US–VA–DLLME) method with a high-performance liquid chromatography–diode array detector was used to analyze UV filters. A bio-derived solvent (i.e., anisole) was used as the extractant in the US–VA–DLLME procedure, along with methanol as the dispersant, a vortexing time of 4 min, and ultrasonication for 3 min. The mass-transfer rate of the extraction process was enhanced due to vortex-ultrasound combination. Various C18 end-capped columns were used to investigate the separation characteristics of the UV filters, with XBridge BEH or CORTECS selected as the separation column. Calibration curves were constructed in the 0.05–5 μg/mL (all filters except octocrylene) and 0.1–10 μg/mL (octocrylene) ranges, and excellent analytical linearities with coefficients of determination (*r*^2^) above 0.998. The developed method was successfully used to analyze sunscreen. Moreover, experiments were designed to simulate the sunscreen-usage habits of consumers, and the cup method was used to extract UV filters from the human stratum corneum. The results suggest that a makeup remover should be employed to remove water-in-oil sunscreens from skin.

## 1. Introduction

Chemical ultraviolet (UV) filters are widely used in a variety of products to protect the skin from UV radiation, with avobenzone (AV) and bis-ethylhexyloxyphenol methoxyphenyl triazine (BEMT) being the most commonly used to protect against UVA (315–400 nm), while octyl methoxycinnamate (OMC), octocrylene (OCT), and ethylhexyl triazone (EHT) are used to protect against UVB (280–315 nm). In addition, the contents of such UV filters in sunscreens are regulated in a number of countries (Table S1) [1,2,3].

Due to their widespread use in cosmetic products and the potential presence of chemical UV filters in the skin, body fluids, and the environment, a range of sample pretreatment methods for chemical UV filters have been developed for analytical purposes [4,5,6,7,8,9]. For example, solid-phase extraction has received growing attention in recent years, but this process requires the use of expensive consumables and specific devices, in addition to being time-consuming and exhibiting a carry-over effect. In contrast, liquid-phase microextraction techniques, such as dispersive liquid–liquid microextraction (DLLME) are inexpensive and offer facile sample preparation, high enrichment levels, and low solvent/sample consumption. A cloudy state is formed in DLLME by rapidly mixing a dispersant and an extractant with an aqueous sample, which increases the contact surface area between the aqueous sample and the extractant [10,11]. Therefore, analyte mass transfer is accelerated, rendering DLLME a more rapid technique than other methods. In addition, various modifications, such as the use of vortexing [12], ultrasound [13,14], or microwave irradiation [13,15] can further increase the analyte mass-transfer rate in DLLME. Indeed, Cinelli et al. developed a new DLLME method that introduces ultrasound and vortexing to improve the phthalate extraction efficiency [16].

Twelve principles of green chemistry have been formulated to protect the environment and human health [17]. As a result, green solvents (such as surfactants, ionic liquids, deep eutectic solvents, and bio-derived solvents) have been used in recent studies as alternatives to the halogenated solvents used as classical DLLME extractants [18,19,20]. Bio-derived solvents are considered to be green, because they are produced in a biorefinery from renewable sources (such as wood, plants, algae, and waste). More specifically, D-limonene is extracted from citrus peel and can be further oxidized to form *p*-cymene [21], whereas anisole is derived from wood lignin [22]. The advantages of such bio-derived solvents include low viscosities, low vapor pressures at room temperature, and good biodegradabilities [20]. Moreover, they are considered as alternatives to classic organic solvents due to their similar physicochemical properties [21]. Although bio-derived solvents offer attractive prospects for reducing environmental issues, the applications of bio-derived solvents remain limited and requires further research.

The percutaneous absorption of chemical UV filters can be assessed by in vitro or in vivo methods [23,24,25], such as those using the Franz diffusion cell and tape stripping, respectively. However, the composition of the receptor fluid used in the Franz diffusion cell and the force used to remove the tape from the skin during tape stripping can significantly affect transdermal absorption and reduce the reliability of the data [26,27]. To overcome these difficulties, Ishiwatari et al. developed a cup method to determine the ability of methylparaben (MP) to penetrate the skin [28]. The cup method is an in vivo technique that uses a glass cup filled with ethanol to extract MP from the stratum corneum of the human forearm after application of MP-containing formulations. This procedure is simple, non-invasive, does not require special devices, and provides reliable human data.

The aim of this study was to develop a simple, highly efficient, and environmentally friendly analytical method based on ultrasound–vortex-assisted DLLME (US–VA–DLLME) followed by high-performance liquid chromatography–diode array detection (HPLC–DAD) for determining the concentrations of chemical UV filters. For this purpose, a bio-derived solvent was employed as the extraction solvent to eliminate harmful environmental effects. The developed method was then used to analyze sunscreen and to determine the amounts of UV filters on or in the skin after sunscreen application. Furthermore, experiments were designed to simulate consumer cleaning and sunscreen-spreading habits, and the cup method was used to obtain extracts of the human stratum corneum. To the best of our knowledge, this is first time that the cup method has been used to extract chemical UV filters from the human stratum corneum.

## 2. Materials and Methods

### 2.1. Reagents and Chemicals

AV (99.5%), BEMT (98.7%), OCT (97%), EHT (>98%), 2-vinylnapthalene (95%) (2-VNT, internal standard), *n*-propyl acetate, and *p*-cymene were purchased from Sigma-Aldrich (St. Louis, MO, USA). OMC, anisole, and *p*-xylene were supplied by Tokyo Chemical Industry (Tokyo, Japan). Methanol (MeOH), ethanol (EtOH), isopropanol (IPP), formic acid, acetonitrile (ACN), and acetone (ACE) were obtained from Merck (Darmstadt, Germany). The deionized water was produced using a Milli-Q Lab system (Bedford, MA, USA). The components of the two homemade sunscreen lotions were received from local cosmetics producers.

Standard stock solutions of each UV filter were prepared in ACE at a concentration of 1 mg/mL (or 2 mg/mL for OCT). The mixed working standard solution of each UV filter was diluted with water to a final ACE:water composition of 1:39 (*v*/*v*). A solution of 2-VNT (100 μg/mL) was prepared in ACN. All prepared solutions were stored in the dark at 4 °C until required for use.

### 2.2. The US–VA–DLLME Procedure

An aliquot (400 μL) of the standard solution (5 μg/mL (all filters except OCT) and 10 μg/mL (OCT)) or the sample was placed in a 1.5-mL tube and MeOH (140 μL, dispersant) and anisole (160 μL, extractant) were added. The tube was vortexed for 4 min and then subjected to ultrasonication for 3 min (QSONICA, Newtown, CT, USA) with an amplitude of 80%. The resulting cloudy solution was then centrifuged at 14,800 rpm for 1 min, and the bottom phase (165 μL) was transferred to another 1.5-mL tube. The 165-μL bottom phase was dried at 55 °C, and the residue was re-dissolved in 2-VNT (20 μL). An aliquot (2 μL) of the resulting sample was then injected into the HPLC–DAD instrument for analysis.

### 2.3. Instrumentation and Conditions

HPLC–DAD analysis was carried out using an Agilent 1260 Series LC system (Palo Alto, CA, USA) equipped with a quaternary pump, a degasser, an autosampler, and a DAD. An XBridge™ BEH C18 analytical column was used (2.5 μm 2.1 × 50 mm; Waters, Milford, MA, USA). Mobile phase A consisted of 0.25% aqueous formic acid, and mobile phase B was ACN. The spectrum of each UV filter is shown in Figure S1. The detection wavelength for OMC, OCT, and EHT was ~300 nm, while the corresponding value for AV and BEMT was ~350 nm, with a flow rate of 0.35 mL/min. The following gradient program was used: 0–1 min, 45–55% B; 1–10 min, 55–70% B; 10–13 min, 70–100% B; 13–25 min, 100% B. The injection volume was 2 μL.

Four kinds of C18 end-capped column were used to determine the chromatographic conditions for the UV filters, including of Chromolith^®^ Performance (2 × 100 mm; Merck, Darmstadt, Germany), ZORBAX 300SB (3.5 μm 2.1 × 100 mm; Agilent, Santa Clara, CA, USA), XBridge BEH (2.5 μm 2.1 × 50 mm; Waters, USA), and CORTECS (2.7 μm 2.1 × 50 mm; Waters, USA).

### 2.4. Method Validation

The limit of detection (LOD) and limit of quantification (LOQ) values of the UV filters were measured by serial dilution of the analytes until the signal-to-noise ratio was greater or equal to three and ten, respectively. Calibration curves constructed from the peak area ratios of the five UV filter signals to the IS signal were employed. The calibration ranges were 0.05–5 μg/mL (all filters except OCT) and 0.1–10 μg/mL (OCT), and the linearities were evaluated by the coefficients of determination (*r*^2^). Three analyte concentrations (0.25, 1.8, and 3.6 μg/mL for all UV filters except OCT; 0.5, 3.6, and 7.2 μg/mL for OCT) were used to validate the intraday (*n* = 6) and interday (*n* = 6) precision and accuracy of the method.

### 2.5. Cosmetics Application

Homemade sunscreen lotions were prepared in a conventional laboratory manner using oil-in-water (o/w) and water-in-oil (w/o) compositions, as outlined in Table S2. More specifically, the oil and water phases were heated separately at 80 °C until all chemicals were completely dissolved. The oil phase was then added to the water phase (for the o/w formulation), or the water phase was added to the oil phase (for the w/o formulation), and the mixture was stirred until an emulsion formed. Each emulsion contained 1% of the required UV filter.

A sample of the homemade sunscreen (12.5 mg) was then weighed and dissolved in ACE/water (50 mL, 1:39, *v*/*v*), and the resulting sample solution was used in the subsequent US–VA–DLLME procedure.

### 2.6. Evaluating the Sunscreen-Removal Efficiency

Five healthy volunteers (three females and two males), with no wounds on their skin and no history of allergies to UV filters or EtOH, participated in the experiment. The study was approved by the Institutional Review Board of Kaohsiung Medical University Chung-Ho Memorial Hospital (KMUH-IRB-20130135). Three washing solutions were used to evaluate the sunscreen-removal efficiency, including water, facial cleanser, and makeup remover. Both the facial cleanser and makeup remover were commercial products. As the facial cleanser was in the form of a paste, it was prepared as a 0.1% (*w*/*v*) aqueous solution for ease of operation.

Prior to each experiment, the forearm of the subject was cleaned with water and dried using a paper towel. A sample of the homemade sunscreen (6.2 ± 0.05 mg, o/w or w/o) was then applied to the flexor surface of the forearm (area: 3.1 cm^2^). After 15 min, a cotton pad (7 × 5 cm) containing the desired cleaning solution (1 mL) was used to clean the test area, and the pad was collected in a tube. The contents of the tube were dried at 55 °C and then re-dissolved in ACE (1 mL). Subsequently, an aliquot (40 μL) of the solution was transferred to a second tube and dried under the conditions described above. The residue was re-dissolved in ACE/water (400 μL, 1:39, *v*/*v*) and used in the subsequent US–VA–DLLME procedure (Section 2.2).

### 2.7. Evaluating the UV Filter Concentration in Human Skin after Spreading a Sunscreen Sample on the Forearm

Ten healthy volunteers (five females and five males) were recruited in this study under the conditions described in Section 2.6. Prior to each experiment, the forearm of the subject was cleaned with water and dried using a paper towel. The procedure employed here is based on a previous study [28] with some modifications. Two sets of experiments were carried out as follows:

#### 2.7.1. Single Applications of Sunscreen to the Skin with Different Exposure Times (0.5, 1, 4, and 8 h)

A 2.0-mg/cm^2^ dose of the homemade sunscreen (w/o) was applied to the flexor surface of the forearm (area: 3.1 cm^2^) for 0.5, 1, 4, or 8 h.

#### 2.7.2. Double Application of the Sunscreen to the Skin (4 + 4 h)

A 2.0-mg/cm^2^ dose of the homemade sunscreen (w/o) was applied to the forearm for 4 h, after which time the same dose of sunscreen was applied to the same tested area for an additional 4 h exposure.

**Figure 1:**
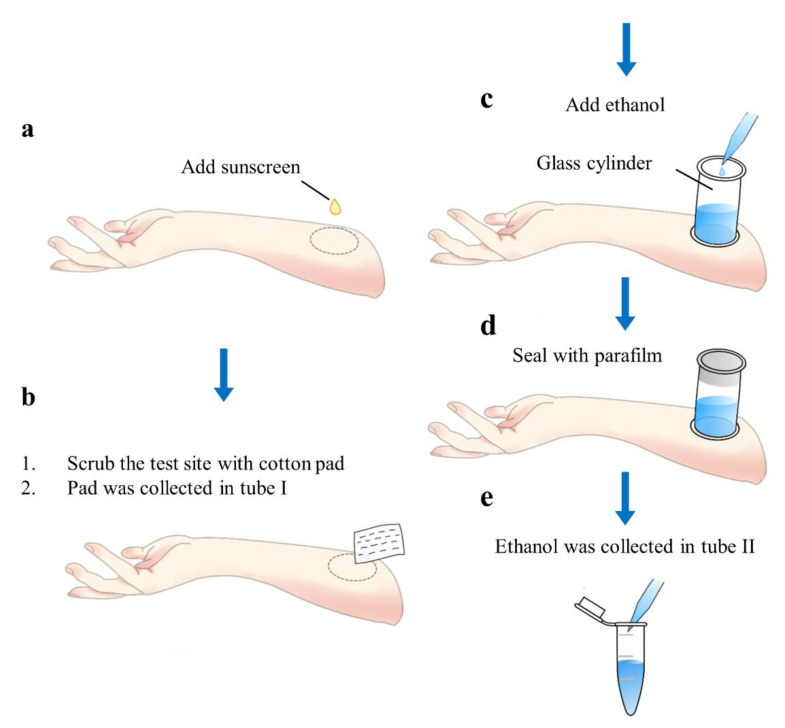
Depicting the cup method.

After exposure for the desired time (0.5, 1, 4, 8, or 4 + 4 h), a cotton pad (7 × 5 cm) containing makeup remover (1 mL) was used to clean the test area (Figure 1a,b). The pad was then collected in a tube (designated tube I), and a glass cylinder (cross-sectional area: 3.1 cm^2^) was used to extract the UV filters from the stratum corneum using EtOH (0.5 mL) (Figure 1c). The open end of the cylinder was sealed with Parafilm (Bemis Flexible Packaging, Neenah, WI, USA) (Figure 1d). After 5 min, the EtOH solution was removed and added to a second tube (designated tube II; Figure 1e). The extraction procedure is represented schematically in Figure 1.

To determine the concentrations of the UV filters on the pad samples, the contents of tube I were dried at 55 °C and re-dissolved in ACE (1 mL). An aliquot (40 μL) of this solution was then removed, added to a new tube, and dried. The residue was re-dissolved in ACE/water (400 μL, 1:39, *v*/*v*), and the resulting solution was used in the subsequent US–VA–DLLME procedure.

To determine the concentrations of the UV filters in the stratum corneum, an aliquot (40 μL) of the EtOH solution from tube II was placed in a new tube and dried at 55 °C. The resulting residue was re-dissolved in ACE/water (400 μL, 1:39, *v*/*v*) and used in the subsequent US–VA–DLLME procedure.

## 3. Results and Discussion

### 3.1. Optimizing the US–VA–DLLME Procedure

The US–VA–DLLME procedure was optimized to maximize both the extraction efficiency and the enrichment factor, with variables including the type and volume of the dispersant and extractant, the vortexing time, and the time and amplitude of the ultrasound treatment.

#### 3.1.1. Effect of the Dispersant

**Figure 2:**
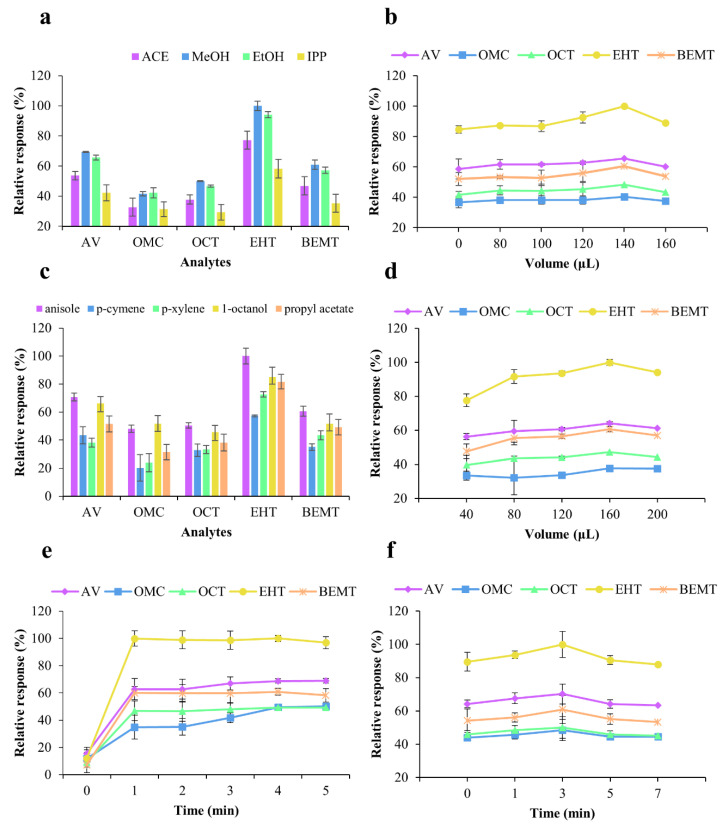
Effects of (**a**) dispersant, (**b**) dispersant volume, (**c**) extractant, (**d**) extractant volume, (**e**) vortexing time, and (**f**) ultrasonication time on the extraction efficiency of the US–VA–DLLME process.

It is important that the dispersant is miscible with the aqueous sample and the extractant to aid in the formation of tiny droplets of the extractant and to increase the extraction efficiency. Hence, four solvents were investigated as possible dispersants, namely ACE, MeOH, EtOH, and IPP. As shown in Figure 2a, MeOH gave the best response for EHT (100% relative response), and, with the exception of OMC, the extraction efficiency of MeOH as the dispersant was greater for all UV filters compared to the those obtained using the other solvents. In addition, the cloudy state was retained for longer when MeOH was employed; consequently, this solvent was selected as the dispersant for the US–VA–DLLME process.

The volume of the dispersant influences both the formation of the cloudy state and the extraction efficiency. As shown in Figure 2b, the best response for EHT (100% relative response) was observed when 140 μL of MeOH was used, with the extraction efficiency being observed to increase as the MeOH volume was increased from 0 to 140 μL. However, the extraction efficiency decreased with further increases in the MeOH volume (to 160 μL), which is likely due to the higher volume resulting in a higher extractant polarity and a lower amount of the UV filter being distributed to the extractant phase. Therefore, a volume of 140 μL was selected for the MeOH dispersant.

#### 3.1.2. Effect of the Extractant

Based on the concept of “like dissolves like,” the extraction efficiency of a UV filter depends on polarity, the number of carbon atoms, the structure, and the presence of functional groups in the extractant. For the purpose of this study, a number of extractants were examined, including aromatics (anisole, *p*-cymene, and *p*-xylene), a long carbon-chain solvent (1-octanol), and an ester (propyl acetate). Importantly, these solvents are both bio-derived and environmentally friendly, as indicated in Sanofi’s solvent selection guide [29].

As shown in Figure 2c, the use of anisole as an extractant resulted in the best EHT response (100% relative response), with similar results being obtained for the other UV filters, with the exception of OMC. Interestingly, the extraction efficiency appeared to depend on the chemical structure. More specifically, anisole gave superior results to the other extractants for the dibenzoylmethane derivative (AV) and triazine derivatives (EHT and BEMT), while similar extraction efficiencies were observed for anisole and 1-octanol for the cinnamate derivatives (OMC and OCT). Although the structures of OMC, OCT, and EHT all contain ester groups, the extraction efficiency of propyl acetate was poor due to its low number of carbon atoms and its low lipophilicity. Therefore, anisole was selected as the extractant for the US–VA–DLLME process.

As shown in Figure 2d, 160 μL of anisole gave the best EHT response (100% relative response). In addition, the extraction efficiency increased as the anisole volume was increased from 40 to 160 μL; hence, a volume of 160 μL was considered to be optimal.

#### 3.1.3. Effect of Vortexing and Ultrasonication

The formation of a cloudy state is the key stage in the DLLME process. In this context, we found that formation of the cloudy state is favored through vortexing and ultrasonication, resulting in enhanced mass transfer of UV filters from the aqueous sample to the extractant.

As shown in Figure 2e, a vortexing time of 4 min gave the best response for EHT (100% relative response). More specifically, the extraction efficiency increased as the vortexing time was increased from 0 to 4 min, with equilibrium being reached at 4 min. A vortex time of 4 min was therefore selected as optimal.

Ultrasonication was used to further reduce the size of the extractant droplets and to increase mass transfer. As shown in Figure 2f, an ultrasonication time of 3 min resulted in the best response for EHT (100% relative response), and the extraction efficiency increased as the ultrasonication time was increased from 0 to 3 min. However, the extraction efficiency decrease when ultrasonication was carried out for more than 3 min, which is possibly ascribable to separation of the extraction solution into two layers during prolonged ultrasonication, thereby reducing the extraction efficiency. It should also be noted here that the ultrasonication energy is proportional to the amplitude. As shown in Figure S2, an ultrasonic amplitude of 100% resulted in the best EHT response (100% relative response), with the extraction efficiency increasing as the ultrasound amplitude was increased from 20% to 80%, while similar extraction efficiencies were observed at both 80% and 100%. Indeed, an amplitude of 80% was optimal for AV, OMC, and OCT; consequently, this amplitude was used in subsequent experiments.

Based on the above results, the optimal conditions for the US–VA–DLLME process were determined to be: 140 μL of MeOH as the dispersant, 160 μL of anisole as the extractant, a vortex time of 4 min, and an ultrasonication time of 3 min, at 80% amplitude. Finally, the anisole (lower) and aqueous (upper) phases separated into two layers, with the UV filters being dissolved in the anisole layer. Figure S3 shows the chromatograms of the same concentrations of UV filters obtained before and after the US–VA–DLLME process, for which enrichment factors of between 11.1 and 17.1 were determined.

Due to the fact that AV (C_20_H_22_O_3_), OMC (C_18_H_26_O_3_), and OCT (C_24_H_27_NO_2_) interact similarly with C18, separating these UV filters is challenging. The separation performance of the UV filters on various C18 end-capped columns under the same gradient conditions (Section 2.3) was investigated; the separation characteristics are listed in Table 1. Although the Chromolith and ZORBAX 300SB columns are both 10 cm in length, AV and OMC were poorly resolved (*R_s_* < 1.5), while the UV filters were well separated on the 5-cm XBridge BEH column (*R_s_* > 1.5); unfortunately, this column has been discontinued, with the CORTECS column being an alternative. The XBridge BEH column has a smaller average particle diameter (*dp*), and *dp*^2^ is proportional to the mass transfer in the mobile phase. Solid-core particles in the CORTECS column improve column performance by lowering the A, B, and C terms in the van Deemter equation. Therefore, these two columns exhibit lower theoretical plate height equivalents (HETPs) and higher column efficiencies. The XBridge BEH and CORTECS columns perform similarly (Table 1).

### 3.2. Method Validation

To determine its applicability, the developed method was validated in terms of its linearity, precision, accuracy, and LOD. The calibration curves showed good linearities, with coefficients of determination (*r*^2^) greater than 0.998 over the 0.05–5 μg/mL (all filters except OCT) and 0.1–10 μg/mL (OCT) calibration ranges. As shown in Table 2, the five UV filters exhibit LODs (*S*/*N* ≥ 3) of 15 ng/mL. The relative standard deviations (RSDs) and the relative errors (REs) of this method during intraday and interday analyses were <4.9%.

Table 3 compares the analytical parameters reported for the determination of UV filters in cosmetics and biological samples. As indicated, solid-phase extraction is the most common pretreatment method used to determine UV filters, which is more expensive and cumbersome than liquid-phase extraction. In addition, the experimental process should minimize the volumes of toxic solvents used to reduce exposure risks and environmental damage according to the principles of green analytical chemistry [17]. Indeed, based on Sanofi’s solvent guide, the extractant solvents used in previous studies are not environmentally friendly [29], while in contrast, we used anisole, a low toxicity and bio-derived solvent. Moreover, the broad linear ranges and low LODs of the developed method render it sufficient for the analysis of cosmetics and biological samples.

### 3.3. Cosmetics Analysis

**Figure 3:**
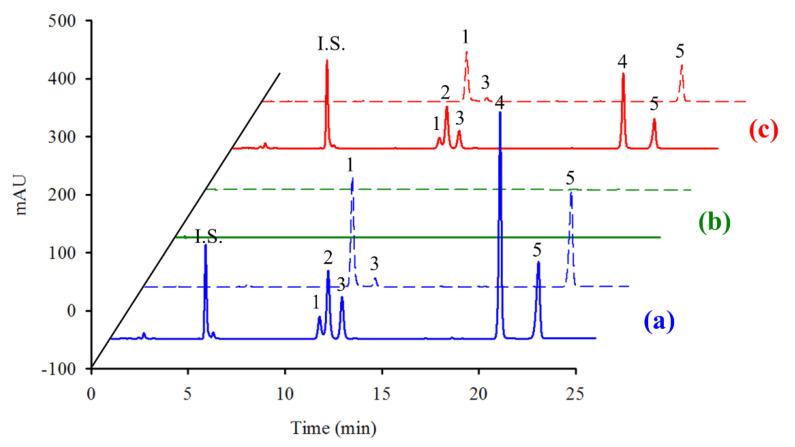
Chromatograms of (**a**) the home-made sunscreen (w/o) (blue), (**b**) the UV filters extracted from the stratum corneum of subject 4 without (green), and (**c**) with the application of home-made sunscreen (w/o) for 0.5 h (red). Detection wavelengths: 300 nm (solid line); 350 nm (dashed line). Peaks: I.S. = 2-VNT, 1 = AV, 2 = OMC, 3 = OCT, 4 = EHT, 5 = BEMT.

The developed method was used to determine the UV filter contents of two home-made sunscreens (o/w and w/o). The obtained chromatograms are shown in Figure 3a, with the determined amounts of UV filters being listed in Table 4, which reveals that the UV filter recoveries range from 87.8% to 104.7%, indicating that the developed method can be used to determine the UV filter contents of such cosmetics.

### 3.4. Evaluating the Concentrations of UV Filter Residues in Human Skin after Exposure to Sunscreen

We designed an experiment to simulate the cleaning and sunscreen-spreading habits of consumers to evaluate the concentrations of UV filters remaining in the skin after sunscreen application. More specifically, we evaluated the sunscreen removal efficiencies using water, facial cleanser, and makeup remover. The removal efficiency of the cleaning solution was determined using the following equation:

(1) $$\mathrm{Removal}\;\mathrm{efficiency}=\frac{\mathrm{the}\;\mathrm{amount}\;\mathrm{of}\;\mathrm{UV}\;\mathrm{filters}\;\mathrm{in}\;\mathrm{the}\;\mathrm{cotton}\;\mathrm{pad}\;\left(\mu g\right)}{\mathrm{the}\;\mathrm{amount}\;\mathrm{of}\;\mathrm{UV}\;\mathrm{filters}\;\mathrm{in}\;\mathrm{the}\;\mathrm{applied}\;\mathrm{sunscreen}\;\left(\mu g\right)}$$

**Figure 4:**
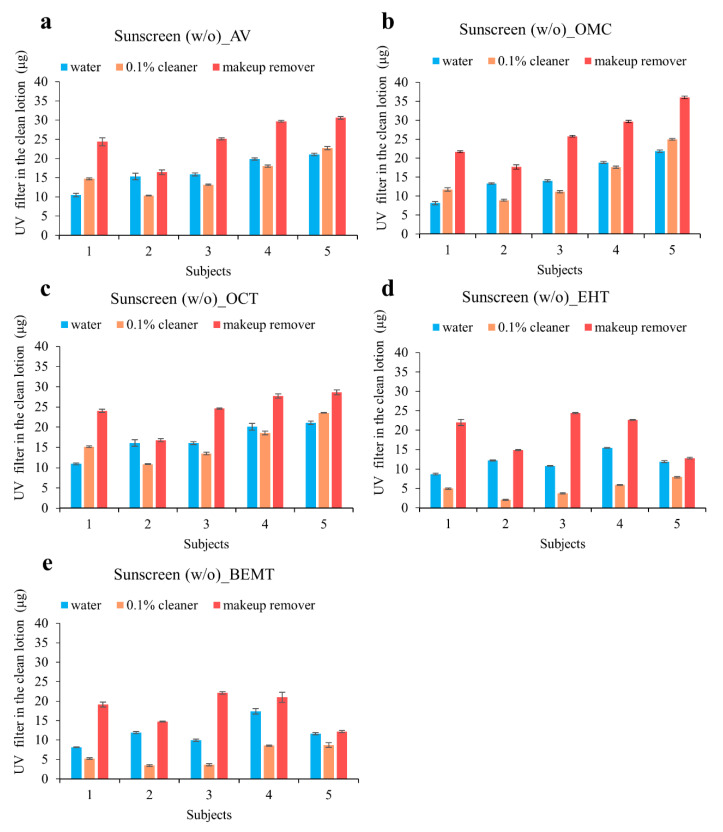
Effects of three cleaning solutions (water, 0.1% facial cleanser, and makeup remover) on the removal of the UV filters of home-made sunscreen (w/o) on the skin of five human volunteers.

Due to the fact that the denominator is the same for the various facial cleaners, this study simply determined the amount of UV filters in the cotton pad to evaluate the removal efficiency of the various cleaning solutions. As shown in Figure 4 and Figure S4, makeup remover most effectively removed the UV filter (especially for the w/o sunscreen), which is likely due to the fact that makeup remover contains greater quantities of surfactants than facial cleanser or water. Makeup remover is therefore recommended for cleaning the skin and to remove any remaining UV filters after sunscreen application. Indeed, the main commercial sunscreens are water-resistant; hence, their removal is challenging without the aid of a surfactant.

**Figure 5:**
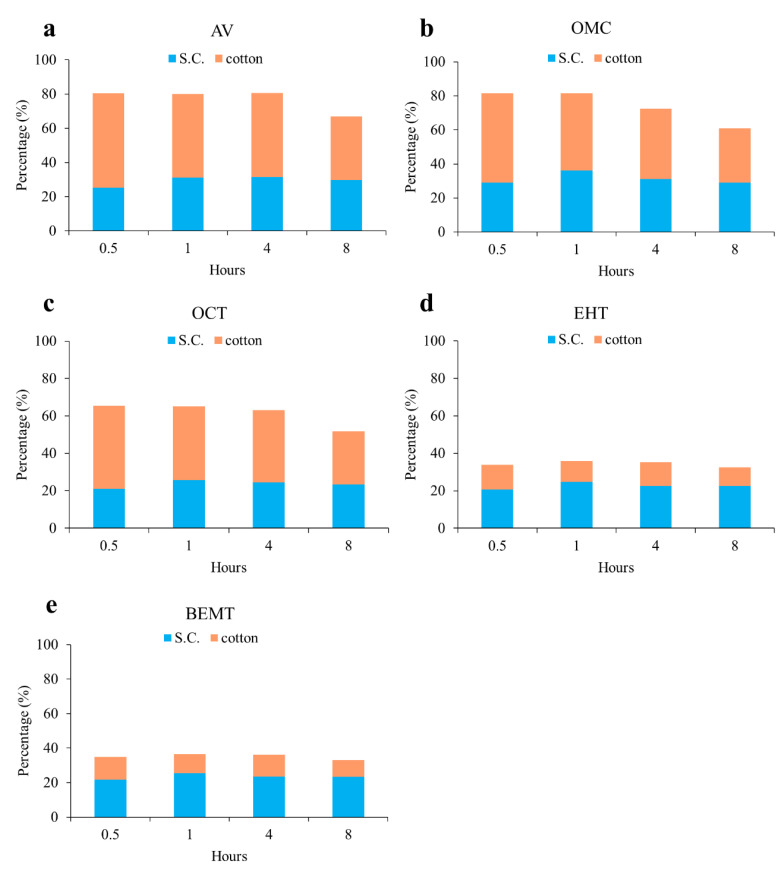
Percentages of UV filters in the cotton (non-penetrated) and the stratum corneum extract after the application of the home-made sunscreen (w/o) on human skin for 0.5, 1, 4, and 8 h of exposure.

To determine the amounts of UV filters accumulated in the skin after sunscreen application, ten healthy volunteers participated in an experiment in which 2 mg/cm^2^ of the home-made sunscreen (w/o) was applied. Chromatograms of the extracts from the stratum corneum of subject 4 are shown in Figure 3b,c, while the quantities of UV filters present in the cotton pad and the stratum corneum following sunscreen application for different periods of time are presented in Figure 5 and Table S3. The UV filters present in the cotton pad are considered to have remained on the skin surface. As shown in Figure 5 and Table S3, the amounts of AV, OMC, and OCT present in the cotton pad were greater than those in the stratum corneum, whereas the opposite was observed for EHT and BEMT, which was attributed to the fact that EHT and BEMT are lipophilic compounds. A w/o-type sunscreen was used in this experiment, which facilitated facile EHT and BEMT penetration into the skin. In addition, the amounts of UV filters present in the stratum corneum reached equilibrium after exposure for 1 h (Figure 5), which is a shorter time than those reported in previous studies [24,30,31] and is likely due to the fact that stratum corneum was analyzed rather than plasma or urine. As shown in Figure 5, the percentages of the determined UV filters in the cotton pad and the stratum corneum did not reach 100% (the initial UV filter value given for the experiment); hence, we speculated that the undetermined UV filters (25.7–69.6%) may have penetrated into deeper skin layers, as UV filters have been reportedly detected in plasma and urine after sunscreen application [30,31,32]. Apart from the use of a w/o formulation, the lipophilicities of EHT and BEMT facilitate their rapid skin penetration and relatively facile transport to plasma.

To ensure that the anti-UV effects of the UV filters are maintained, the reapplication of sunscreen to the skin at regular intervals is recommended. Therefore, we also compared the amounts of UV filters present in the stratum corneum after single and double sunscreen applications. As shown in Table 5, the amounts of UV filters were determined to be 1.6–1.8-fold higher after double application than after a single application, which is consistent with the results of a previous study [25]. It should also be noted that due to differences in human skin conditions, it is normal for the RSD values in Table 5 to be higher than 10%; this has also been reported in previous studies [30].

## 4. Conclusions

Herein, we reported the development of a simple, sensitive, and environmentally friendly method for determining five ultraviolet (UV) filters in cosmetics and the human stratum corneum through the combination of ultrasound–vortex-assisted dispersive liquid–liquid microextraction (US–VA–DLLME) and high-performance liquid chromatography–diode array detection to analyze the UV filters. The introduction of vortexing and ultrasonication was shown to improve mass transfer in the DLLME process, thereby improving detection limits. In addition, anisole, a bio-derived solvent, was employed as the extraction solvent to eliminate any harmful effects on the environment. Furthermore, we used the cup method to extract the UV filters from the human stratum corneum. The cup method overcomes the disadvantages of the common skin-penetration test, and the obtained data are more representative of human skin. In this study, the cup method was successfully used to determine the contents of five UV filters in the human stratum corneum after the topical application of sunscreen. We found that the UV filter contents in the stratum corneum increased as the number of applied doses increased, with equilibrium being reached after 1 h. On the basis of our results, we recommend the use of makeup remover to remove sunscreen from the skin. Moreover, the developed method can be combined with LC-MS/MS to determine the amounts of UV filters in plasma and urine samples in the future.

## Figures and Tables

**Table 1:** Separation characteristics of the UV filters on different columns.

	Chromolith				ZORBAX 300SB				XBridge BEH				CORTECS			
Chemistry	C_18_				C_18_				C_18_				C_18_			
I.D. (mm)	2				2.1				2.1				2.1			
L (mm)	100				100				50				50			
Particle Size (µm)	-				3.5				2.5				2.7			
UV filters	*k*	*α*	*N*/*m*	*R_s_*	*k*	*α*	*N*/*m*	*R_s_*	*k*	*α*	*N*/*m*	*R_s_*	*k*	*α*	*N*/*m*	*R_s_*
AV	13.5		56,878		12.7		237,759		23.5		543,055		27.7		749,816	
		1.0		0.2		1.0		0.9		1.0		1.5		1.0		1.6
OMC	13.7		73,457		13.0		352,883		24.5		389,656		28.9		478,847	
		1.1		0.9		1.1		3.4		1.1		2.0		1.0		2.6
OCT	14.3		79,834		14.0		405,038		26.0		416,811		30.8		603,501	
		-		-		-		-		-		-		-		-
EHT	25.0		357,860		25.5		1,500,064		42.4		1,174,803		55.5		1,805,575	
		-		-		-		-		-		-		-		-
BEMT	28.7		316,880		26.8		881,338		47.3		928,022		62.8		837,478	

**Table 2:** Analytical parameters for the proposed UV filter analysis method.

UV Filters	Linear Range (µg/mL)	Determination Coefficient (*r*^2^)	LOD (ng/mL)	Interday (%, *n* = 6) ^a^						Intraday (%, *n* = 6) ^a^					
				Q1		Q2		Q3		Q1		Q2		Q3	
				RSD	RE	RSD	RE	RSD	RE	RSD	RE	RSD	RE	RSD	RE
AV	0.05–5	0.999	15	1.9	3.3	2.8	−4.0	1.2	−1.9	1.6	0.2	0.4	1.8	0.8	2.2
OMC	0.05–5	0.998	15	4.9	0.2	4.2	−2.7	3.9	1.1	1.8	0.6	1.6	0.2	1.4	0.3
OCT	0.1–10	0.999	15	2.8	−0.8	3.1	−2.6	1.2	−1.5	1.4	0.5	0.8	2.5	0.3	0.8
EHT	0.05–5	0.999	15	3.4	−1.3	3.0	−2.0	1.5	−1.6	1.5	−2.0	1.2	−1.0	0.3	−0.4
BEMT	0.05–5	0.999	15	3.9	−1.8	3.1	−2.1	1.2	−1.5	2.2	−1.3	0.8	−1.7	1.4	−0.2

^a^ The concentrations of Q1, Q2, and Q3 for UV filters (except OCT) were 0.25, 1.8, and 3.6 µg/mL, respectively. The concentrations of Q1, Q2, and Q3 for OCT were 0.5, 3.6, and 7.2 µg/mL, respectively.

**Table 3:** Analytical methods for chemical UV filters in cosmetics and biological samples.

Instrumental Method	Pretreatment Method	Sample	Solvent	Linear Range (ng/mL)	LOD (ng/mL)	Analyte	Ref.
GC–MS	SPME	Fish tissue		1000–7000 ^a^	5–25 ^a^	OMC, OCT	[4]
HPLC–MS/MS	PLE	Cosmetics	MeOH	1–1000	10–31 ^a^	AV, OMC, OCT	[5]
HPLC–MS/MS	UAE–dSPE	Human placenta tissue	ACN	0.3–15 ^a^	0.1 ^a^	OMC, OCT	[6]
HPLC–MS/MS	QuEChERS–reverse SPE	Aquatic invertebrates	ACN	0.1–25	2.0–3.3 ^a^	OMC, OCT	[7]
HPLC–DAD	SLE	Porcrine skin	DMF, EtOH	10,000–50,000	280–1400	BEMT, EHT	[8]
HPLC–DAD	dilution	Cosmetics	MeOH, EA	1800–250,000	32–67	AV, OCT	[9]
HPLC–DAD	US–VA–DLLME	Cosmetics, extracts of the human stratum corneum	MeOH, anisole	50–5000; 100–10,000 ^b^	15	AV, OMC, OCT, EHT, BEMT	This study

Abbreviation: SPME, solid phase microextraction; PLE, pressurized liquid extraction; UAE, ultrasound assisted extraction; dSPE, dispersive solid phase extraction; SLE, solid liquid extraction; DMF, dimethylformamide. ^a^ ng/g ^b^ Linear range for UV filters (except OCT) and OCT, respectively.

**Table 4:** UV filter contents of home-made sunscreens (o/w and w/o).

UV Filters	o/w ^a^			w/o ^a^		
	Found (μg/mL)	RSD (%)	Recovery (%)	Found (μg/mL)	RSD (%)	Recovery (%)
AV	2.2	2.4	88.1	2.2	0.8	87.8
OMC	2.5	2.3	100.2	2.5	2.6	100.2
OCT	2.4	2.4	95.7	2.6	2.6	104.7
EHT	2.5	3.3	99.4	2.5	3.7	99.4
BEMT	2.5	5.4	100.6	2.5	2.3	98.4

^a^ The known concentration of UV filters in home-made sunscreen sample solutions were 2.5 μg/mL.

**Table 5:** UV filter contents of cup-method samples after single or double applications of the home-made sunscreen (w/o) for 8 h (subjects = 10).

UV Filters	Single				Twice			
	Cotton		S.C.		Cotton		S.C.	
	Mean [Range, μg]	RSD (%)	Mean [Range, μg]	RSD (%)	Mean [Range, μg]	RSD (%)	Mean [Range, μg]	RSD (%)
AV	18.5 [8.6–37.4]	46.0	14.7 [6.1–32.2]	57.3	39.4 [22.2–49.9]	20.5	23.6 [12.4–53.9]	52.8
OMC	18.0 [8.3–32.9]	48.8	16.4 [7.4–27.9]	41.0	42.2 [21.8–61.2]	26.6	30.0 [16.1–47.8]	46.2
OCT	17.2 [8.9–29.6]	38.0	14.1 [6.0–26.1]	49.6	38.3 [22.8–50.6]	21.2	23.6 [12.0–53.9]	53.6
EHT	5.8 [2.6–12.9]	61.1	13.4 [5.3–27.6]	51.3	18.2 [5.3–34.6]	57.1	22.4 [10.6–43.5]	44.7
BEMT	5.6 [2.6–12.0]	58.7	13.3 [5.7–28.1]	52.4	18.2 [5.5–33.2]	56.6	21.9 [11.1–41.5]	42.8

## Supplementary Materials

The following are available online: Table S1: UV filter structures and regulations, Table S2: UV filter contents of cup-method samples after application of the homemade sunscreen (w/o) for 0.5, 1, 4, and 8 h, Table S3: Compositions of the homemade sunscreens, Figure S1: Spectra of UV filters, Figure S2: Effect of ultrasonication amplitude on the extraction efficiency of the US–VA–DLLME process, Figure S3: Chromatograms of mixed standard solutions without (blue) and with extraction (red) at 5 μg/mL (except OCT) and 10 μg/mL (OCT). Detection wavelengths: 300 nm (solid line); 350 nm (dash line). Peaks: ACE, 1 = AV, 2 = OMC, 3 = OCT, 4 = EHT, 5 = BEMT, Figure S4: Effects of three cleaning solutions (water, 0.1% facial cleanser, and makeup remover) on the removal of the UV filters of the homemade sunscreen (o/w) on the skins of five human volunteers.
