# Supplementary material for: Ultrasound–Vortex-Assisted Dispersive Liquid–Liquid Microextraction Combined with High Performance Liquid Chromatography–Diode Array Detection for Determining UV Filters in Cosmetics and the Human Stratum Corneum
Source: Molecules. 2020 Oct 12;25(20):4642. doi: 10.3390/molecules25204642 (PMC7587185; doi:10.3390/molecules25204642)
Supplement: Supplementary file 1 [file molecules-25-04642-s001.pdf]

Supporting Information

Ultrasound–Vortex-Assisted Dispersive Liquid–Liquid Microextraction Combined with High Performance Liquid Chromatography–Diode Array Detection for Determining UV Filters in Cosmetics and the Human Stratum Corneum

Fang-Yi Liao [1], Yu-Lin Su [2], Jing-Ru Weng [3], Ying-Chi Lin [1,4] and Chia-Hsien Feng [2,4,5,6,*]

[1] School of Pharmacy, College of Pharmacy, Kaohsiung Medical University, Kaohsiung 80708, Taiwan; pulu9@hotmail.com (F.-Y.L.); yclin@kmu.edu.tw (Y.-C.L.)
[2] Department of Fragrance and Cosmetic Science, College of Pharmacy, Kaohsiung Medical University, Kaohsiung 80708, Taiwan; aitian897@gmail.com
[3] Department of Marine Biotechnology and Resources, National Sun Yat-sen University, Kaohsiung 80424, Taiwan; jrweng@mail.nsysu.edu.tw
[4] The Ph.D. Program in Toxicology, College of Pharmacy, Kaohsiung Medical University, Kaohsiung 80708, Taiwan
[5] Institute of Medical Science and Technology, National Sun Yat-sen University, Kaohsiung 80424, Taiwan
[6] Department of Medical Research, Kaohsiung Medical University Hospital, Kaohsiung 80708, Taiwan
* Correspondence: chfeng@kmu.edu.tw; Tel.: +886-7-3121101-2805

Table of Contents

Table S1. UV filter structures and regulations…………………………………………………………... 3

Table S2. Compositions of the homemade sunscreens……………………...……………………………………5

Table S3. UV filter contents of cup-method samples after application of the homemade sunscreen (w/o) for 0.5, 1, 4, and 8 h………………………………………………………………………………. 6

Figure S1. Spectra of the UV filters. ………..……………..……………………………….……….... 7

Figure S2. Effect of ultrasonication amplitude on the extraction efficiency of the US–VA–DLLME process. ……..………………………………..…………………….…..…. 8

Figure S3. Chromatograms of mixed standard solutions without (blue) and with extraction (red) at 5 μg/mL (except OCT) and 10 μg/mL (OCT). Detection wavelengths: 300 nm (solid line); 350 nm (dash line). Peaks: ACE, 1=AV, 2=OMC, 3=OCT, 4=EHT, 5=BEMT. …………………………………………………………………….... 9

Figure S4. Effects of three cleaning solutions (water, 0.1% facial cleanser, and makeup remover) on the removal of the UV filters of the homemade sunscreen (o/w) on the skins of five human volunteers. ……………………………………………………….……………… 10

Table S1. UV filter structures and regulations.

Types	Analytes	Structure	log P	Limit Concentration (%)		
				EU [1]	TFDA [2]	FDA [3]
UVA	AV	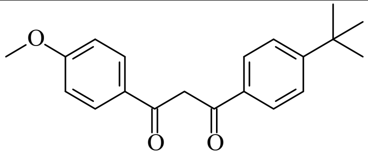	4.8	5	5	3
	BEMT	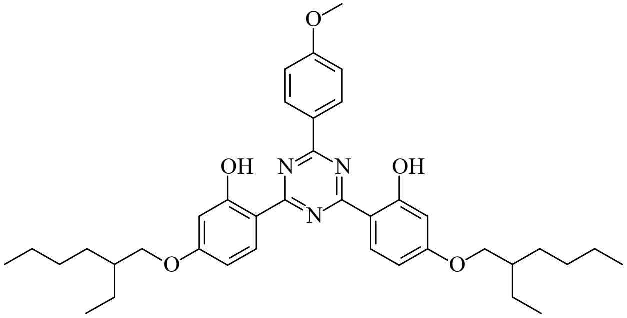	10.4	10	10	NA
UVB						
	OMC	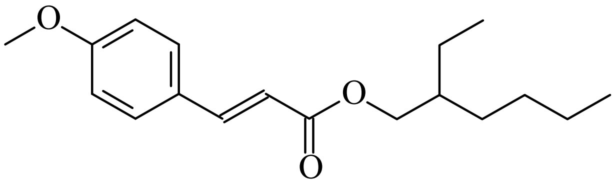	5.3	10	10	7.5
	OCT	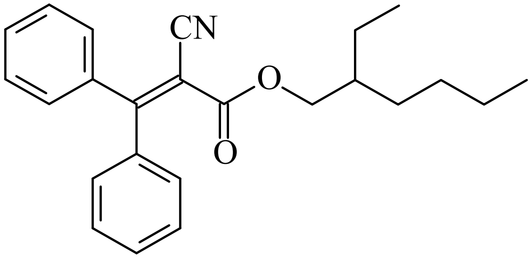	7.1	10	10	10

EHT	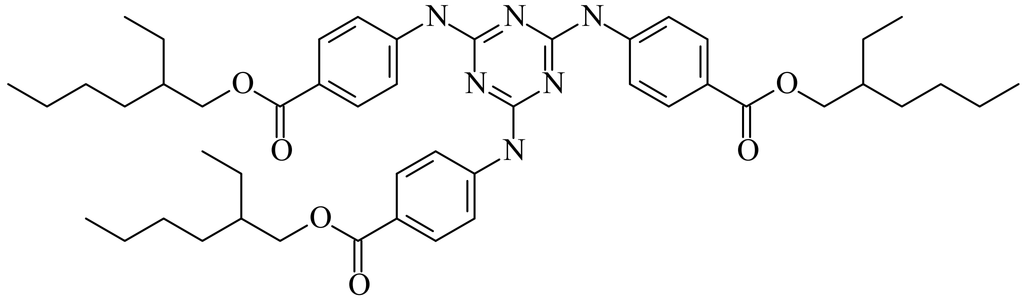	14.5	5	5	NA

Abbreviations: Not approved (NA). The log P values of listed UV filters were referenced from *https://pubchem.ncbi.nlm.nih.gov*

Table S2. Compositions of the homemade sunscreens.

Ingredients	% (w/w) of ingredient	
	o/w	w/o
**Oil phase**		
Cetyl alcohol	0.5	0.5
Stearic acid	2	2
Mineral oil (Paraffinum Liquidum)	3.5	
Olea Europaea (olive) oil	2.5	
Avobenzone (AV)	1	1
Ethylhexyl methoxycinnamate (OMC)	1	1
Octocrylene (OCT)	1	1
Ethylhexyl triazone (EHT)	1	1
Bis-Ethylhexyloxyphenol Methoxyphenyl Triazine (BEMT)	1	1
Beeswax		0.5
Cyclopentasiloxane		2
Glycerol monostearate		2
**Aqueous phase**		
Propylene glycol	5	
Potassium hydroxide	0.1	
Glycerin		5
Sodium borate		0.3
Deionized water	81.4	82.7

Table S3. UV filter contents of cup-method samples after application of the homemade sunscreen (w/o) for 0.5, 1, 4, and 8 h.

UV filters	0.5 h		1 h		4 h		8 h	
	Cotton	S.C.	Cotton	S.C.	Cotton	S.C.	Cotton	S.C.
	Mean [Range, µg] (RSD, %)	Mean [Range, µg] (RSD, %)	Mean [Range, µg] (RSD, %)	Mean [Range, µg] (RSD, %)	Mean [Range, µg] (RSD, %)	Mean [Range, µg] (RSD, %)	Mean [Range, µg] (RSD, %)	Mean [Range, µg] (RSD, %)
AV	27.4 [20.1-32.2] (13.9)	12.6 [5.3-16.7] (31.4)	24.3 [17.3-30.3] (15.1)	15.5 [9.7-25.2] (29.8)	24.4 [10.3-41.4] (33.3)	15.7 [9.7-37.8] (51.9)	18.5 [8.6-37.4] (46.0)	14.7 [6.1-32.2] (57.3)
OMC	29.6 [18.7-37.7] (18.8)	16.5 [6.7-25.3] (33.5)	25.7 [17.0-36.6] (23.7)	20.4 [13.4-32.6] (31.4)	23.4 [6.5-38.7] (37.5)	17.6 [10.4-33.2] (36.7)	18.0 [8.3-32.9] (48.8)	16.4 [7.4-27.9] (41.0)
OCT	26.8 [20.2-29.9] (12.7)	12.7 [5.4-17.2] 30.8	23.9 [17.4-29.2] (13.2)	15.4 [9.8-24.6] (28.7)	23.3 [10.5-33.2] (26.3)	14.8 [9.8-28.6] (35.9)	17.2 [8.9-29.6] (38.0)	14.1 [6.0-26.1] (49.6)
EHT	7.7 [3.4-13.3] (45.8)	12.3 [5.0-17.7] (34.8)	6.6 [3.0-18.3] (74.9)	14.7 [8.4-25.7] (36.1)	7.4 [2.7-20.2] (72.0)	13.4 [7.6-19.3] (25.1)	5.8 [2.6-12.9] (61.1)	13.4 [5.3-27.6] (51.3)
BEMT	7.5 [3.4-12.8] (45.0)	12.4 [5.0-17.4] (34.1)	6.4 [3.2-16.8] (68.9)	14.5 [8.8-25.1] (34.9)	7.2 [2.7-18.7] (68.6)	13.4 [8.2-21.0] (27.2)	5.6 [2.6-12.0] (58.7)	13.3 [5.7-28.1] (52.4)

Abbreviation: S.C., stratum corneum.

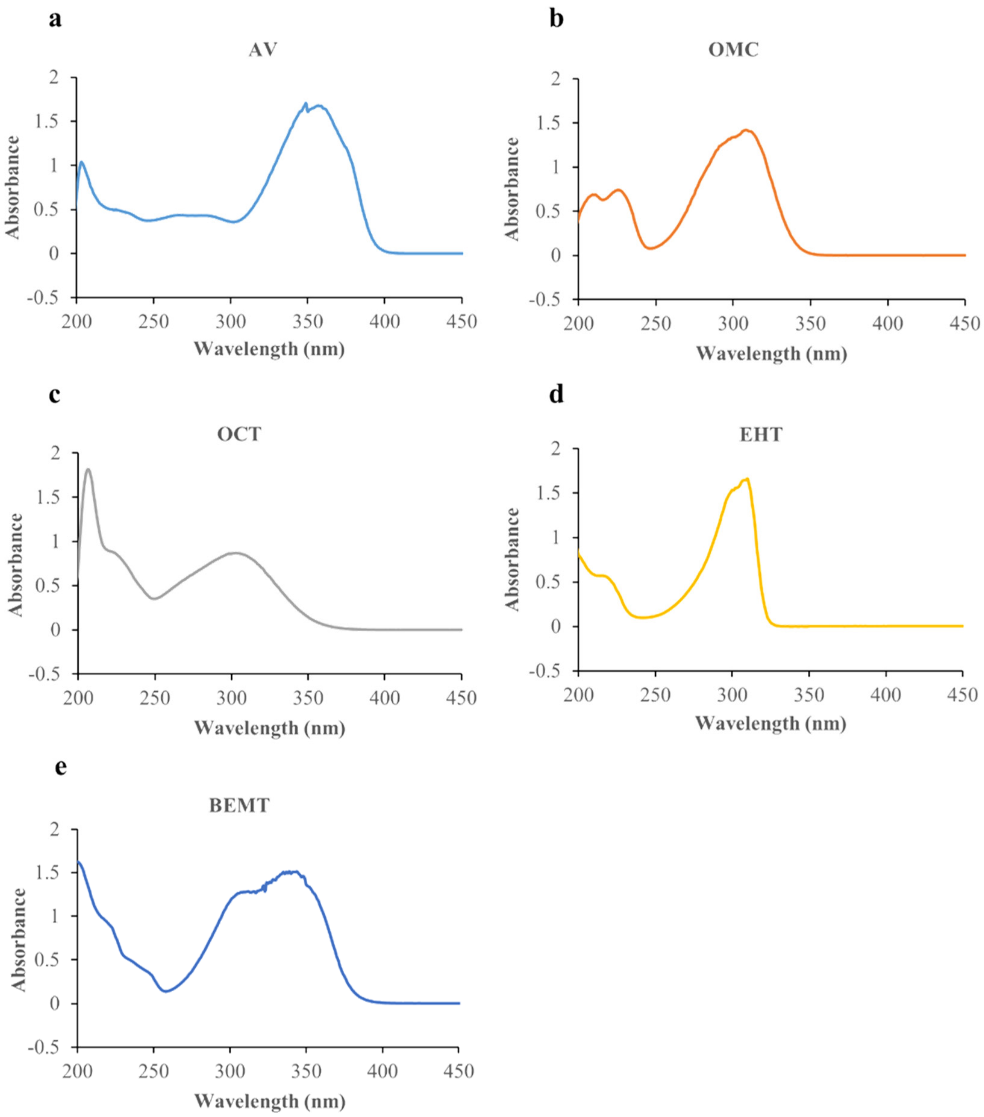

Figure S1. Spectra of the UV filters.

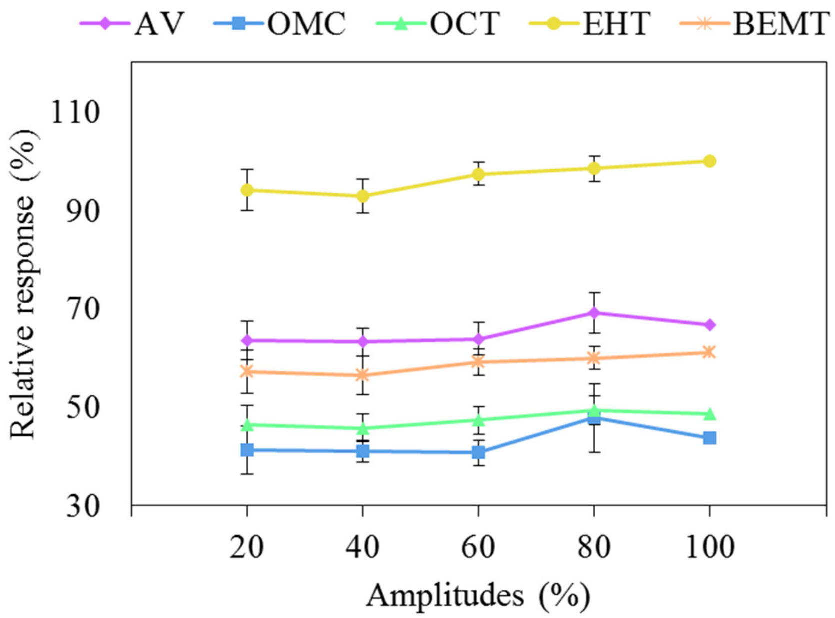

Figure S2. Effect of ultrasonication amplitude on the extraction efficiency of Table 3. Chromatograms of mixed standard solutions without (blue) and with extraction (red) at 5 μg/mL (except OCT) and 10 μg/mL (OCT). Detection wavelengths: 300 nm (solid line); 350 nm (dash line). Peaks: ACE, 1 = AV, 2 = OMC, 3 = OCT, 4 = EHT, 5 = BEMT.

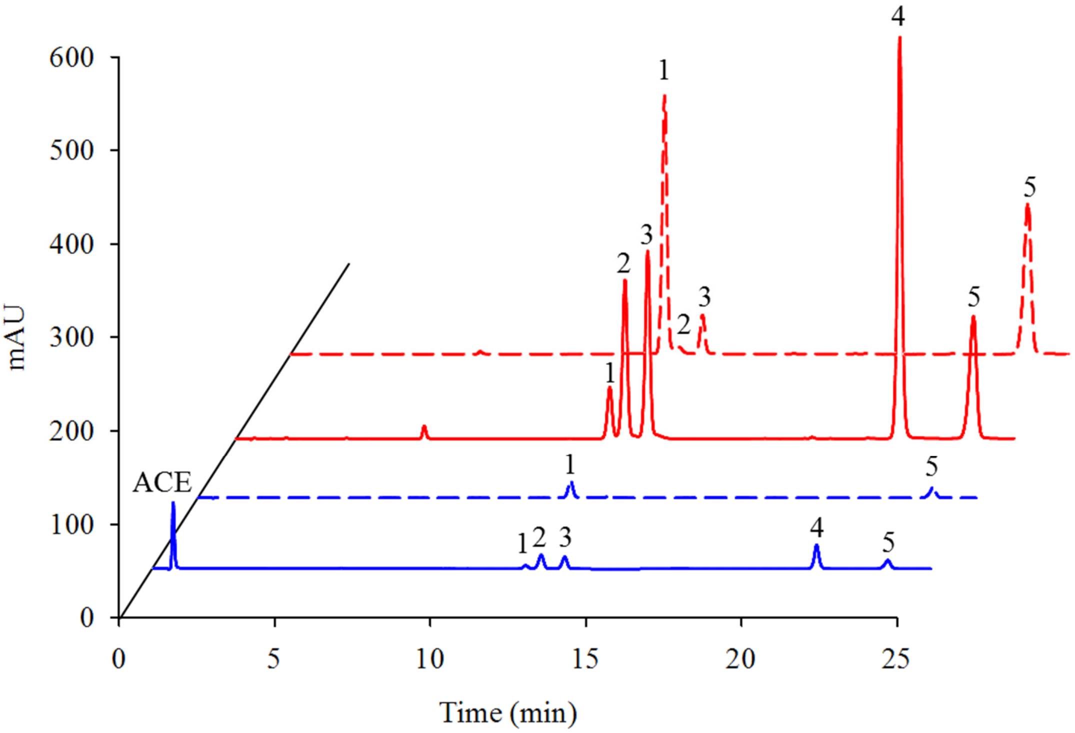

Figure S3. Chromatograms of mixed standard solutions without (blue) and with extraction (red) at 5 μg/mL (except OCT) and 10 μg/mL (OCT). Detection wavelengths: 300 nm (solid line); 350 nm (dash line). Peaks: ACE, 1=AV, 2=OMC, 3=OCT, 4=EHT, 5=BEMT.

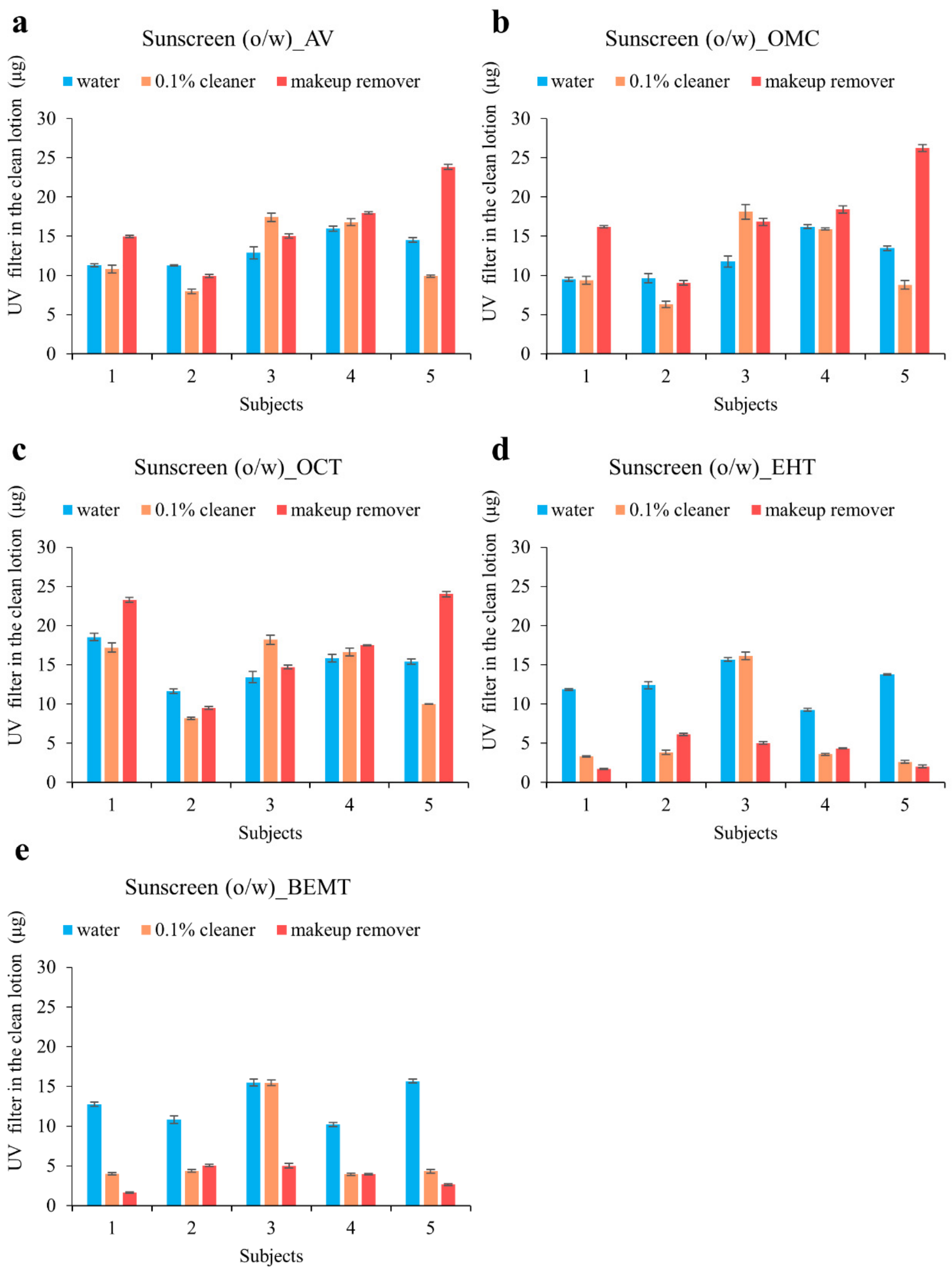

Figure S4. Effects of three cleaning solutions (water, 0.1% facial cleanser, and makeup remover) on the removal of the UV filters of the homemade sunscreen (o/w) on the skins of five human volunteers.
